# The *Bacillus cereus* Hbl and Nhe Tripartite Enterotoxin Components Assemble Sequentially on the Surface of Target Cells and Are Not Interchangeable

**DOI:** 10.1371/journal.pone.0076955

**Published:** 2013-10-18

**Authors:** Inka Sastalla, Rasem Fattah, Nicole Coppage, Poulomi Nandy, Devorah Crown, Andrei P. Pomerantsev, Stephen H. Leppla

**Affiliations:** Microbial Pathogenesis Section, Laboratory of Parasitic Diseases, National Institute of Allergy and Infectious Diseases, National Institutes of Health, Bethesda, Maryland, United States of America; The University of Texas-Houston Medical School, United States of America

## Abstract

*Bacillus cereus* is a spore-forming, Gram-positive bacterium commonly associated with outbreaks of food poisoning. It is also known as an opportunistic pathogen causing clinical infections such as bacteremia, meningitis, pneumonia, and gas gangrene-like cutaneous infections, mostly in immunocompromised patients. *B. cereus* secretes a plethora of toxins of which four are associated with the symptoms of food poisoning. Two of these, the non-hemolytic enterotoxin Nhe and the hemolysin BL (Hbl) toxin, are predicted to be structurally similar and are unique in that they require the combined action of three toxin proteins to induce cell lysis. Despite their dominant role in disease, the molecular mechanism of their toxic function is still poorly understood. We report here that *B. cereus* strain ATCC 10876 harbors not only genes encoding Nhe, but also two copies of the *hbl* genes. We identified Hbl as the major secreted toxin responsible for inducing rapid cell lysis both in cultured cells and in an intraperitoneal mouse toxicity model. Antibody neutralization and deletion of Hbl-encoding genes resulted in significant reductions of cytotoxic activity. Microscopy studies with Chinese Hamster Ovary cells furthermore showed that pore formation by both Hbl and Nhe occurs through a stepwise, sequential binding of toxin components to the cell surface and to each other. This begins with binding of Hbl-B or NheC to the eukaryotic membrane, and is followed by the recruitment of Hbl-L_1_ or NheB, respectively, followed by the corresponding third protein. Lastly, toxin component complementation studies indicate that although Hbl and Nhe can be expressed simultaneously and are predicted to be structurally similar, they are incompatible and cannot complement each other.

## Introduction

*Bacillus cereus* is a Gram-positive, spore-forming bacterium. It is ubiquitously distributed in the environment but can also colonize the human and invertebrate intestines [1], [2]. Genetically, *B. cereus* is closely related to *B. thuringiensis*, an insect pathogen, and *B. anthracis*, the causative agent of anthrax [3]. In European agriculture, *B. cereus* spores are frequently added as a probiotic to animal feed for fattening purposes [4], and the use of some strains as biological control agents to reduce fungal growth on crops has also been suggested [5]. In humans, *B. cereus* is associated with usually self-limiting foodborne diseases that are caused by contamination of a variety of foods such as rice, meat, spices, milk, and pasta [6]. Furthermore, in hospitals, *B. cereus* is increasingly recognized as an opportunistic pathogen and as the cause of local and systemic infections such as bacteremia, cellulitis, meningitis, and endophthalmitis [7], some of which can occur in immunocompromised patients and neonates [7], [8].

A number of exotoxins contribute to the pathogenicity of *B. cereus* in both gastrointestinal and other infections. While emetic food-poisoning symptoms have been attributed to intoxication with the heat- and gastric acid-resistant peptide cereulide, diarrheal symptoms are mainly caused by the three pore-forming enterotoxins cytolysin K (CytK), non-hemolytic enterotoxin (Nhe), and hemolysin BL (Hbl) [9]. Expression of the latter three toxins is controlled by a quorum-sensing system involving the global regulator PlcR and the processed peptide PapR [10]; this system regulates the expression of more than 40 proteins, some of which are known virulence factors and proteases [11]. CytK is a single-protein toxin that was identified in a strain associated with a severe food poisoning outbreak in France [12]. Nhe and Hbl are both tripartite pore-forming toxins that require the combined action of the three proteins NheA, NheB, and NheC, or Hbl-B, Hbl-L_1_, and Hbl-L_2_, respectively. Nhe was isolated from the supernatant of a strain that caused a large food-poisoning outbreak in Norway in 1995 [13], and Hbl was first identified as a protein causing dermonecrosis [14]. Hbl is also an important contributor to the development of endophthalmitis [15]. Being predicted to have similar modes of action and structures, Hbl and Nhe also share significant sequence homology [16]. Two studies [17], [18] found Hbl to be expressed in 42–73% and Nhe in 97–99% of food-poisoning associated strains, and Hbl expression appears less frequent in non-pathogenic isolates [17]. Thus, while nearly all strains express Nhe, Hbl is produced by only a subset. Furthermore, the simultaneous expression of both tripartite toxins was documented in some strains [17], [18].

The exact modes of action of the Hbl and Nhe toxins are poorly understood. The proposed cellular binding moiety of Hbl is the 38.5-kDa protein Hbl-B [19], although it was later suggested that Hbl-L_1_ and Hbl-L_2_ can bind independently to erythrocytes [20]. Similarly, it has been reported that several of the Nhe subunits interact with the eukaryotic cell surface. In 2004, using Western blotting, Lindbäck *et al.* found NheB but not NheC or NheA to bind to lysed Vero cells [21]. In a later study, however, the authors identified NheB binding using one assay but not another; additionally, NheC was described as a binding moiety [22]. Additionally, it was recently shown that in solution, NheB and NheC form complexes, and it was suggested that this complex, in addition to NheC alone, may bind to the cellular surface [23]. Thus, for both the Hbl and Nhe toxins, the binding order of toxin components and the mechanisms of pore formation are not clear.

Structurally, Hbl-B has a striking resemblance to HlyE/ClyA/SheA, a single-component hemolytic enterotoxin of the gram-negative bacteria *Escherichia coli*, *Salmonella enterica*, and *Shigella flexneri* [24]. This similarity is also predicted for the Nhe cellular binding proteins NheB and NheC [16], indicating that both gram-positive and gram-negative bacteria may have convergently evolved a similar pore-forming toxin. HlyE is mainly composed of alpha helix bundles but also contains a short hydrophobic 20-amino acid sequence that is arranged in two antiparallel beta sheets [25], [26]. This hydrophobic region is predicted to insert into the cellular membrane, causing structural rearrangements and oligomerization into a dodecameric pore [27]. Mutagenesis studies of HlyE have further confirmed that the hydrophobic region is crucial for pore-forming activity [28]. Because of the structural similarity, it is likely that the binding proteins of the Nhe and Hbl toxins function in a similar manner.

In this study we identified Hbl toxin as the major cytotoxic protein secreted by *B. cereus* ATCC 10876, and we describe the high-yield expression and purification of Hbl and Nhe toxin components in an avirulent strain of *B. anthracis.* We further provide data supporting the hypothesis that pore formation by both Nhe and Hbl is achieved by stepwise, ordered binding of single components rather than simultaneous binding of multiple components, and we show that although the toxins have sequence (and presumably structural) similarities, they cannot complement each other.

## Materials and Methods

### Ethics

All animal experiments were reviewed and approved by the Animal Care and Use Committee of the National Institute of Allergy and Infectious Diseases, National Institutes of Health, and performed according to NIH and Animal Welfare Act guidelines.

### Bacterial Strains, Culture Conditions, and Preparation of Supernatants

*Bacillus cereus* ATCC 10876 was grown in Brain Heart Infusion (BHI, BD Biosciences) broth at 37°C and 225 rpm (for toxicity studies) or in Luria-Bertani medium (for mutagenesis purposes). The PlcR isogenic mutant [29] was grown in the presence of erythromycin (10 µg/ml). The Hbl isogenic mutant strain was generated by a Cre-*loxP* based mutagenesis approach [30], [31]. Briefly, two fragments representing the upstream and downstream region of the Hbl-1 locus were amplified using primers Hbl-LF1 and Hbl-LF2, and Hbl-RF1 in combination with Hbl-RF2, respectively (Table S1), and separately cloned into plasmid pSC containing two *loxP* recognition sites, resulting in pSC_HblF1 and pSC_HblF2, respectively. *B. cereus* ATCC 10876 was first transformed with pSC_HblF1, and colonies with single-crossover chromosomal integration of the plasmid were selected on erythromycin plates at 37°C. Plasmid pCrePAS, which contains the Cre recombinase gene under control of the *B. anthracis* protective antigen promoter, was introduced, and colonies having the pSC plasmid backbone excised by Cre-recombinase were selected by erythromycin sensitivity. Next, plasmid pSC-HblF2 was introduced, followed by selection of chromosomal plasmid integration and repeated pCrePAS introduction. This mutagenesis procedure caused the final deletion strain to be antibiotic sensitive and left a single loxP site instead of the *hbl* locus. For toxicity assays, bacteria grown to desired optical densities were centrifuged (4,000×g), the culture supernatants were sterile-filtered (0.22 µm, Millipore, Billerica, MA), and stored at −80°C.

### Eukaryotic Cells and Toxicity Studies

RAW264.7 macrophages (ATCC, Manassas, VA) and HT1080 fibroblasts (ATCC) were cultured in Dulbecco’s Modified Eagle Medium (DMEM, Invitrogen, Carlsbad, CA) containing 10% fetal bovine serum, 10 mM HEPES, and 100 µg/ml gentamycin. Chinese Hamster Ovary (CHO) WTP4 cells [32] were cultured in Minimal Essential Medium Alpha (AMEM) supplemented with 5% fetal bovine serum, 10 mM HEPES, and 100 µg/ml gentamicin. Human neutrophils were isolated from fresh heparinized blood obtained from the NIH Blood Bank using a Histopaque 1119/1077 (Sigma, St. Louis, MO) double gradient sucrose isolation procedure as recommended by the manufacturer. This purification procedure yielded approximately 10^7^ neutrophils per 6 ml of blood. Isolated neutrophils were cultured in RPMI (Invitrogen) medium. All cells were grown at 37°C in a 5% CO_2_ atmosphere.

For cytotoxicity studies, serial dilutions of sterile bacterial culture supernatants or medium were incubated with eukaryotic cells grown in a 96-well plate. For some experiments, cells were incubated with dilutions (for Hbl-neutralization studies) or with a set concentration of 5% (for Nhe neutralization studies) mouse serum containing antibodies raised against Hbl-B, L_1_, L_2_, or with pre-immune serum for 30 min before the addition of supernatants. After 90 min, supernatants were aspired and cell viability was determined by addition of 3-(4,5-Dimethylthiazol-2-yl)-2,5-diphenyltetrazolium bromide (MTT, Sigma) at a final concentration of 1 mg/ml. Cells were incubated for an additional 45 min, the medium was removed, and cells were lysed in 50 µl dissolving buffer (0.5% SDS, 25 mM HCl, 90% isopropanol). Viability was quantified at 590 nm. Viability of non-adherent cells was determined using the Celltiter 96® Aqueous One Solution Cell Proliferation Assay kit (Promega, Madison, WI). For complementation toxicity studies and for Nhe neutralization studies, CHO WTP4 cells were incubated in a 96-well plate with mixtures of Hbl and Nhe toxin components (5 nM for Hbl toxins, 3% final concentration of recombinant Nhe toxin-containing sterile culture supernatants) for 30–45 min, the supernatant was aspired, and viability was assessed by MTT staining.

For priming studies, 100 µl of confluently grown CHO cells were primed with one of the purified (for Hbl, 5 nM of each component) or BH460 secreted, non-purified (for Nhe, 1∶50 of each component) toxin components for 30 min at 37°C and 5% CO_2_, and excess and unbound toxin components removed by 5 washes with AMEM medium. Cells were challenged by addition of the two complementary components and incubated for 30 min. Cell viability was assessed by MTT as described above. PBS or two-toxin-component treated samples served as negative, and samples receiving all three toxin proteins served as positive controls.

### Mouse Immunizations

Balb/cJ mice (Jackson Laboratories) (n = 3/group) were immunized subcutaneously with 500 µl containing 10 µg of either Hbl-B, Hbl-L_1_, or Hbl-L_2_, 250 µl of Alum (Sigma) and 200 µl PBS. At 2 and 5 weeks after the first injection, mice were boosted via the same route with 10 µg of antigen in a final volume of 100 µl. All animals were anaesthetized by inhalational administration of isoflurane and terminally bled 9 weeks after the first antigen injection by cardiac puncture.

### Intraperitoneal Toxicity Studies and Analyses

Mice (DBA/2J, 8–14 weeks of age, n = 3/group) (Jackson Laboratory, Bar Harbor, ME) were injected in the peritoneum with 500 µl of sterile *B. cereus* culture supernatants or with heat-treated supernatants (all diluted 1∶5). After 5 or 45 min, mice were sacrificed by CO_2_ asphyxiation and cells were isolated from the peritoneal cavity by washes with 12 ml of PBS containing 0.5% bovine serum albumin (PBSA). Cells were concentrated and washed by centrifugation (1000×g for 10 min). Red blood cells (RBCs) were eliminated by treatment with ammonium chloride lysing buffer (ACK, Invitrogen), and cells were resuspended in 200 µl of PBSA. One µl of propidium iodide (1 mg/ml) (Invitrogen) was added immediately before flow cytometric analysis using an LSRII (BD Biosciences, San Jose, CA). Data analyses were performed using FlowJo version 7 (Tree Star Inc., Ashland, OR).

### Hbl Expression Analyses in *B. cereus* ATCC 10876

Bacteria were grown in BHI and supernatants were harvested at the desired growth phases, sterile-filtered, and separated on a 4–20% Tris/glycine denaturing gels. Proteins were blotted onto nylon membrane (Invitrogen) and incubated with mouse serum (1∶2000) containing antibodies against Hbl-B, L_1_, or L_2_ in LI-COR Blocking Solution (LI-COR Biosciences, Lincoln, NE). An anti-mouse IR-800 antibody (1∶5000) (Rockland Immunochemicals, Gilbertsville, PA) was used as secondary antibody. Membranes were imaged with an Odyssey Infrared Scanner (LI-COR Biosciences).

### Supernatant Fractionation and Mass Spectrometry

Five hundred ml of BHI sterile *B. cereus* sterile supernatants were concentrated approximately 33-fold using a 10,000 MW cut-off membrane (Millipore), filtered through a 0.22 µm filter (Millipore) and applied to a Sephacryl S-100 HR column (Pharmacia, Kalamazoo, MI) using 1 × PBS as the mobile phase. Aliquots of protein-containing fractions were collected at 4°C and 10 µl of these were added to 90 µl of RAW264.7 macrophages grown in 96-well plates. Fractions that were able to kill macrophages in 60 min were pooled and protease inhibitor 4-(2-aminoethyl)-benzenesulfonylfluoride (AEBSF, 2 µg/ml) (US Biological, Swampscott, MA) was added before dialysis overnight at 4°C against 2 L of 5 mM HEPES. The dialyzed suspension was further separated on a Q Sepharose FF column (GE Healthcare, Piscataway, NJ) with a 0–500 mM NaCl gradient in 20 mM Tris HCl (pH 8.0). Single or combined fractions were tested for cytotoxic activity on RAW264.7 macrophages as described in the section on eukaryotic cells and toxicity studies, and biologically active fractions were concentrated using 10,000 MW cut-off membranes (Millipore) to approximately 1 ml, separated on a 4–20% Tris/Glycine gel, stained with Coomassie, and bands of interest were excised and analyzed by MS/MS sequencing at the Protein Chemistry Research Technology Branch of the National Institute of Allergy and Infectious Disease.

### Cloning, Protein Expression and Purification

Hbl and Nhe toxin components (including the DNA sequence of the predicted signal peptide) were amplified from *B. cereus* ATCC 10876 genomic DNA and cloned via *NdeI*/*BamHI* restriction sites into the expression vector pSW4, which allowed for the *B. anthracis* protective antigen promoter-controlled expression of genes [33]. We further added a histidine tag to the C-terminal end of all proteins. All primer sequences are listed in Table S1. Sequence-verified constructs were passed through the Dam/Dcm methylation-deficient *E. coli* strain SCS110 (Agilent, Santa Clara, CA) and used to transform *B. anthracis* strain BH460 [34]. Secreted toxin proteins were expressed by growth of BH460 containing pSW4-constructs overnight in 2–5 liters of FA broth [35] supplemented with 20 µg/ml kanamycin. AEBSF (4 µg/ml) (US Biological) was added to the cultures before centrifugation and sterile filtration using a 0.22 µm filter. Proteins in the supernatants were adsorbed batch-wise to Phenyl-Sepharose Fast Flow resin (low substitution, GE Healthcare) by addition of solid ammonium sulfate to 2 M final concentration. The resins were washed with 1.5 M ammonium sulfate in 10 mM Tris-HCl, 1 mM EDTA (both pH 8.0), and the proteins eluted in the same buffer containing 0.3 M ammonium sulfate. Approximately 450 ml of eluate was collected and proteins were precipitated by addition of 30 g solid ammonium sulfate per 100 ml eluate. The resulting precipitate was collected by centrifugation at 18,370×g for 20 min. Hbl and Nhe proteins were dissolved in 50 ml of 20 mM Tris HCl, 5 mM EDTA (pH 8.0) and dialyzed for 5 h against 10 mM Tris HCl, 0.5 mM EDTA (pH 8.0).

#### Purification of Hbl proteins

After dialysis, Hbl proteins were loaded onto a column containing Q-Sepharose Fast Flow resin (GE Healthcare) that was pre-equilibrated with a buffer containing 20 mM Tris HCl and 0.5 mM EDTA (pH 8.0). Toxin proteins were eluted with a NaCl gradient from 0 to 0.5 M at a flow rate of 1 ml/min. Protein-containing fractions were identified by SDS-Phast Gel (GE Healthcare) analysis. Next, toxin-containing fractions were dialyzed as before and loaded onto a hydroxyapatite column (Bio-Rad, Hercules, CA) that was pre-equilibrated with 0.02 M potassium phosphate, 0.1 M NaCl buffer (pH 7.0). Proteins were eluted at 1 ml/min in a 0.02–1.0 M (for Hbl-L_1_ and Hbl-L_2_) or 0.02–0.5 M (for Hbl-B) potassium phosphate gradient, protein-containing fractions were analyzed by SDS-Phast gel analysis (GE Healthcare) and dialyzed overnight against 2 L of 10 mM HEPES and 0.5 mM EDTA (pH 7.5). Hbl-B and Hbl-L_2_ were concentrated using a Centricon 30,000 Da cut-off membrane (Millipore). All Hbl proteins were frozen and stored at −80°C. Final protein yields for Hbl toxin per liter culture were 48.5 mg (Hbl-L_1_), 75.5 mg (Hbl-L_2_) and 37.5 mg (Hbl-B).

#### Purification of Nhe proteins

Proteins were loaded onto a Q-Sepharose Fast Flow column (GE Healthcare) that was pre-equilibrated with a buffer containing 20 mM ethanolamine (MP Biomedical, Santa Ana, CA), 2% (w/v) ethylene glycol, 2 mM CaCl_2_, and 0.5 mM EDTA, (pH 9.0, pH 9.5 for NheB). Protein elution was performed in the same buffer with a gradient of NaCl from 0–0.5 M. For NheA, we observed some precipitate after dialysis against 10 mM HEPES, 0.5 mM EDTA, 25 mM NaCl; most of the proteins resolubilized after the addition of NaCl to a final concentration of 250 mM. Because of the high yield (33.6 mg per liter of culture) and pureness of the protein, no further purification was required. For NheB, which was also very pure after the ion-exchange column, we continuously encountered loss of protein activity with further purification steps; thus, we stopped further purification. We further purified NheC. Fractions of the Q-sepharose eluate containing NheC were pooled and concentrated using a Centricon 30,000 Da cut-off membrane (Millipore) to a volume of 10 ml. Because we observed two protein bands in SDS Phase gels, we employed size fractionation with a Sephacryl S-200 HR column (Pharmacia). Fractions containing protein of the size predicted for NheC were pooled and concentrated to a final volume of 9 ml. The final yield of NheC and NheB was 2.2 mg and 41.8 mg per liter of culture, respectively. All Nhe toxins were stored in 50% glycerol at −20°C.

Masses and purity of all proteins were verified by liquid chromatogram-electrospray mass spectrometry using an HP/Agilent 1100 MSD instrument (Hewlett Packard, Palo Alto, CA) at the core facility of the National Institute of Diabetes and Digestive and Kidney Diseases (Bethesda, MD).

### Biotinylation of Nhe Proteins

Recombinantly expressed and purified Nhe subunits were incubated for 4 h at room temperature with biotin-N-hydroxysuccinimide ester (Sigma) at a molar ratio of 1∶100. Biotinylated proteins were dialyzed for 4 h against a buffer containing 20 mM Tris-HCl and 100 mM NaCl. The concentration of the retrieved proteins was determined using the BCA Protein Assay Kit (Thermo Scientific, Waltham, MA). Biotinylated proteins were stored at −20°C.

### Toxin Binding Studies and Confocal Microscopy

CHO cells were seeded onto coverslips (12 mm in diameter) (VWR, Radnor, PA) and grown to semi-to-full confluence. All toxin components were used at a dilution of 5 µg/ml. For Hbl binding studies, cells were incubated with single recombinant toxin proteins for 30 min at 37°C and 5% CO_2_. Cells were washed 3×with PBS, fixed with 3.7% formaldehyde in PBS (for single component binding), or incubated with a second toxin component for an additional 30 min before PBS washes and fixation. For Nhe binding studies, cells were incubated with biotinylated Nhe proteins (for single toxin binding detection) or with non-biotinylated NheC (for sequential binding studies) for 30 min at 37°C and 5% CO_2_. Cells were washed 3×with PBS and either fixed as above, or incubated with biotinylated NheB or NheA for an additional 30 min before washing and fixation. For immunofluorescence, coverslips with fixed cells were incubated with 1% BSA in PBS for 1 h. All washes were performed with PBS containing 0.05% Tween−20 between incubation steps. To Hbl-treated cells, mouse sera containing antibodies raised against Hbl-B, Hbl-L_1_, or Hbl-L_2_ (1∶400 in 1% BSA, 1 h) were added, followed by washes and treatment with anti-mouse Alexa Fluor 594 antibodies (1∶200 in 1% BSA, 1 h) (Invitrogen). Biotinylated Nhe proteins were detected by incubation with Alexa Fluor 488 labeled streptavidin (1∶300 in 1% BSA, Invitrogen). DAPI (Invitrogen) was added at a concentration of 1∶2000. Coverslips were mounted onto glass slides using PermaFluor (Thermo Fisher Scientific, Waltham, MA) and sealed. Microscopy was performed with a Leica SP5 confocal microscope with a 488 argon and a 594 HeNe laser. Pictures were taken with a 63×oil objective with a zoom of 2.5. Confocal microscopy was performed by the Imaging Core Facility of the National Institute for Allergy and Infectious Diseases (Bethesda, MD), analyzed using the Leica Application Suite Advanced Lite program (Leica Microsystems, Buffalo Grove, IL), and processed in Adobe Photoshop CS5 Version 12.1 (Adobe, San Jose, CA).

### Statistics

Differences between groups were analyzed in GraphPad Prism (version 5, Graph Pad Software Inc., La Jolla, CA) by using one-way ANOVA followed by a Tukey’s post-test for multiple sample variance, or by using unpaired t-test for sample variance of two samples.

## Results

### *B. cereus* Secreted Cytotoxin(s) Act Species- and Cell Type Independently

**Figure 1:**
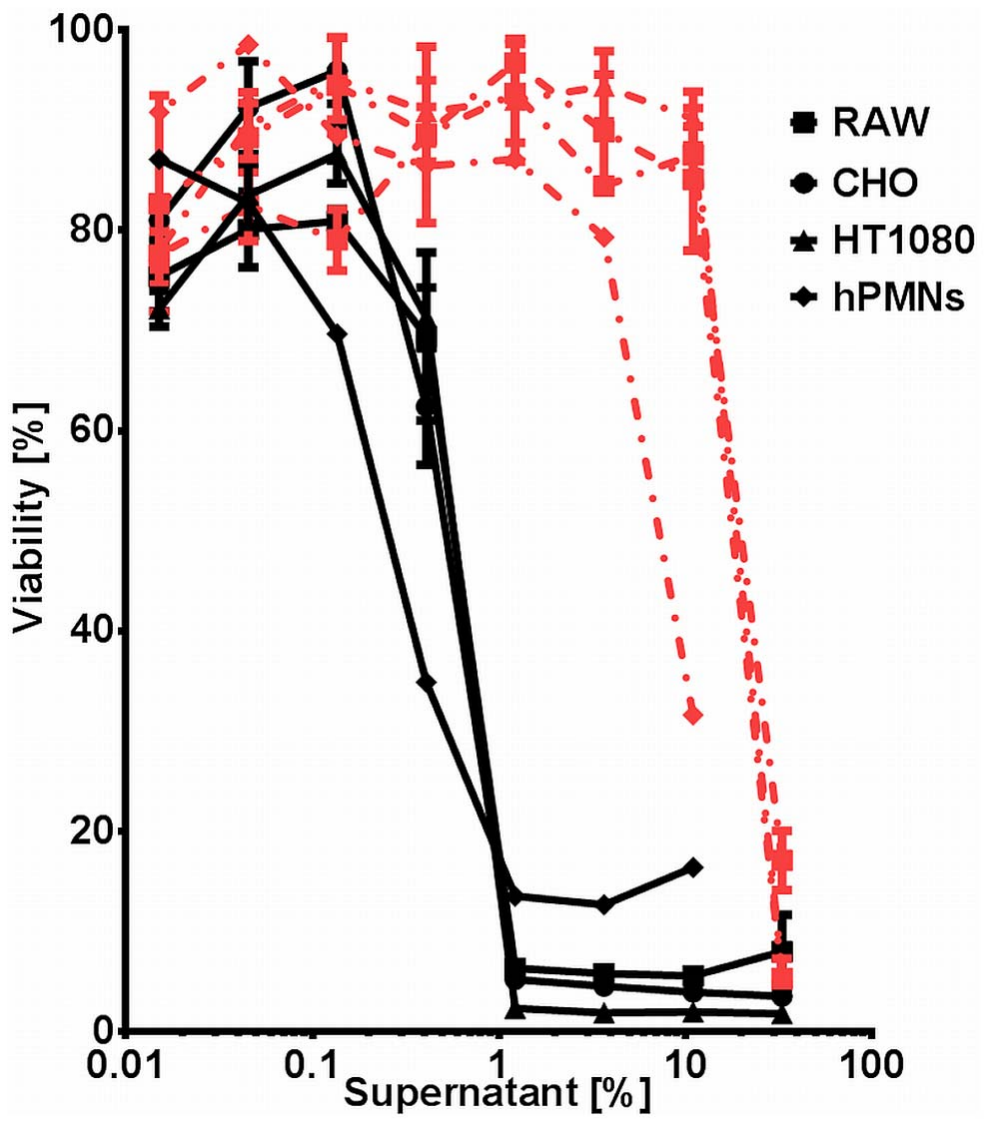
Cytotoxicity of proteins secreted by *B. cereus* ATCC 10876. Cells of different type and origin were challenged for 90*B. cereus* ATCC 10876 (solid lines) or an isogenic mutant defective in the expression of the global regulator PlcR (dotted lines). Viability was assessed by the ability of cells to convert 3-(4,5-Dimethylthiazol-2-yl)-2,5-diphenyltetrazolium bromide to purple formazan and is expressed as % of the untreated control.

During an earlier study aimed at determining cytotoxicity of secreted proteins of different *Bacillus* strains, we observed that exposure of eukaryotic cells to culture supernatants of *B. cereus* ATCC 10876 induced rapid cell death [36]. To determine whether the observed cell toxicity was cell- and species specific, we assessed viability of human fibroblasts (HT1080) and neutrophils (hPMNs), hamster ovary cells (CHO), and mouse macrophages (RAW264.7) in response to dilutions of *B. cereus* culture supernatants. All four tested cell types were highly susceptible to the toxin(s), with a half effective concentration (EC50) of approximately 0.5% of sterile culture supernatant (Figure 1). In contrast, cells challenged with supernatants derived from an isogenic strain defective in the expression of the global regulator PlcR [29] remained viable at concentrations as high as 11%. Higher concentrations killed the cells, indicating the presence of toxin(s) whose expression is independent of PlcR (Figure 1). These results show that *B. cereus* secretes PlcR-regulated toxin(s) that cause rapid cell death *in vitro*.

### *B. cereus* Secreted Proteins have Cytotoxic Effects *in vivo*

**Figure 2:**
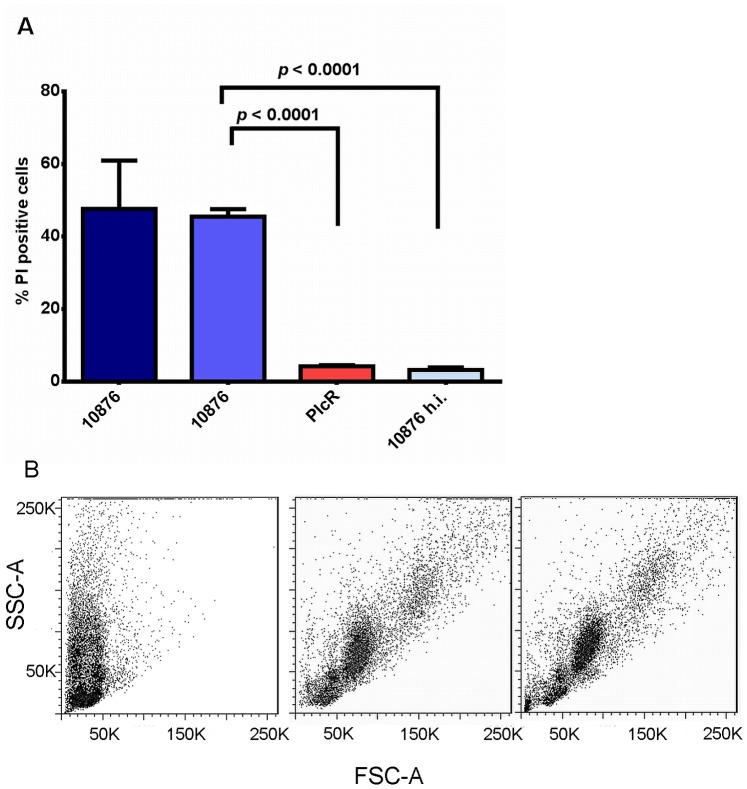
Viability of mouse peritoneal cells after challenge with *B. cereus* ATCC 10876 secreted proteins. (A) Flow cytometric assessment of the percentage of propidium iodide-stained cells isolated from the peritoneal cavity of mice (n = 4/group) injected with 500 µl of diluted supernatants of *B. cereus* ATCC 10876 or *B. cereus* PlcR mutant, or with heat-treated supernatants of *B. cereus* 10876. Bars labeled with “10876” represent 10 min (dark blue) and 45 min (lighter blue) incubation times after injection. (B) Representative forward vs. side scatter plots of cells retrieved from the peritoneal cavity of mice 45 min after injection with supernatants of *B. cereus* ATCC 10876 (left panel), the *B. cereus* PlcR mutant (middle panel), or heat-treated supernatants of *B. cereus* 10876 (right panel).

To investigate the toxic activity of secreted proteins of *B. cereus* in an *in vivo* model, we injected filter sterilized bacterial culture supernatants into the mouse peritoneal cavity, which contains high numbers of immune cells, such as B-cells, T-cells, and naïve macrophages. By analyzing remaining viable cell populations by flow cytometry, we found that as early as 10 min after injection of supernatants, approximately 50% of isolated cells were propidium-iodide (PI) - positive (Figure 2A), and in conjunction with their smaller size (i.e., decreased forward scatter, Figure 2B), these results indicated that the cells were non-viable. The percentage of dead cells did not increase significantly with longer incubation times (Figure 2A). In contrast, cells isolated from animals having received heat-inactivated *B. cereus* supernatants (control) or supernatants derived from an isogenic strain defective in expression of the global regulator PlcR [29] were viable (Figure 2A and 2B). These results show that *B. cereus* secretes PlcR-regulated protein toxin(s) that cause a rapid cell death in an *in vivo* intraperitoneal toxicity model.

### Identification of Hemolysin BL as the Toxicity-inducing Factor

**Figure 3:**
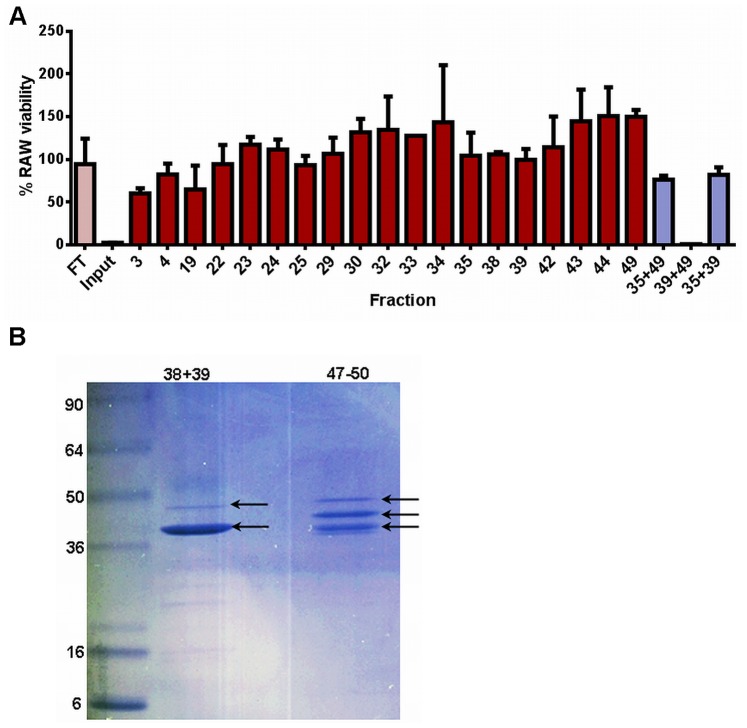
Identification of the toxic components present in *B. cereus* ATCC 10876 culture supernatants. (A) RAW264.7 macrophage toxicity measurement of protein-containing *B. cereus* ATCC 10876 culture supernatant fractions eluted from anion exchange column. FT, flow through fraction; Input, unfractionated supernatant. (B) Coomassie-stained gel of combined, toxic anion exchange fractions. Arrows indicate bands subjected to mass spectrometric analysis.

We used a biochemical approach to identify the secreted *B. cereus* toxin(s) responsible for *in vitro* toxicity. Proteins present in bacterial culture supernatants were separated by size-exclusion and ion exchange chromatography, followed by activity tests of eluates on RAW264.7 macrophages. Surprisingly, we observed cytotoxicity only when certain fractions were combined, while single fractions were nontoxic (Figure 3A). Thus, the purified protein toxin was multi-factorial. Electrophoretic separation of the two pools containing the toxic fractions (Figure 3B) followed by mass spectrometric analysis of the separated bands showed components of the Hbl toxin to be present in the two fractions that were needed for toxicity (Figure 3B). Thus, it appeared likely that the cytotoxicity observed *in vitro* was caused by Hbl.

### *B. cereus* ATCC 10876 Contains Two Copies of the *hbl* Genes

**Figure 4:**
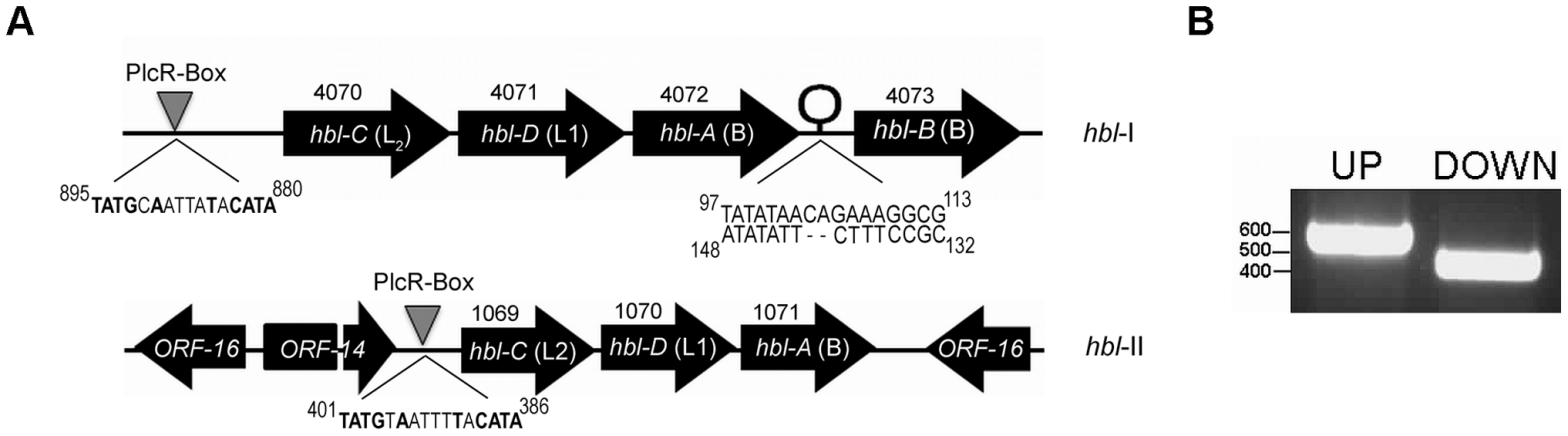
*B. cereus* ATCC 10876 harbors two copies of Hbl-encoding genes. (A) Schematic operon overview (not to scale) of the two hemolysin BL loci in the genome of *B. cereus* ATCC 10876. The PATRIC gene annotation numbers (4070–4073 and 1069–1071) are indicated above the genes, and the PlcR-boxes are shown below, with conserved nucleotides of the *plcR* consensus sequence indicated in bold. Locus *hbl*-I harbors a second copy of an *hbl-A*-like gene, *hblB*, located 375 nucleotides downstream of *hblA*. An inverted repeat representing a putative transcriptional stop site 96 nucleotides downstream of *hblA* is indicated. Up- and downstream of the *hbl* genes in locus *hbl*-II the genes ORF-14 and ORF-16 having homology to the *Bacillus anthracis* plasmid pXO1 replication system are shown. (B) Presence of the *hbl*-II locus in *B. cereus* ATCC 10876 verified by PCR. The first fragment extended from 438 bp upstream of *hblC2* to 85 bp into the coding region of the same gene (total length of the predicted amplicon: 523 bp); the second fragment started at bp 85 in the coding region of *hblA2* and extended 318 bp downstream of the operon (total length of the predicted amplicon: 403 bp).

The three Hbl proteins Hbl-L_2_, Hbl-L_1_, and Hbl-B are encoded by the genes *hblC*, *hblD*, and *hblA*, respectively. We analyzed the *hbl* genes of *B. cereus* ATCC 10876 using the PathoSystems Resource Integration Center (PATRIC) website (www.patricbrc.org). Interestingly, we found that this strain harbors two *hbl* copies in its genome. We termed these two loci *hbl*-I and *hbl*-II, and the corresponding gene numbers are VBlBacCer12024_4070–4072, and VBlBacCer12024_1069–1071, respectively, encoding for the Hbl proteins L_2_, L_1_, and B (Figure 4A). We identified perfect PlcR binding sites 895 (for *hbl*-I) and 401 nucleotides (for *hbl*-II) upstream of the *hblC* (encoding for Hbl-L_2_) start codons and a stem loop structure downstream of the *hbl-A* gene of the *hbl*-I locus, representing a putative transcriptional stop (Figure 4A). Additionally, the *hbl*-I locus contains a fourth gene encoding for an Hbl-B-like protein; this gene, which has been identified in other *B. cereus* strains and named *hbl-B* [37], is 252 nucleotides larger than *hbl-A.* Since it is located after the stem loop structure, it may not be co-transcribed with the other three toxin genes, consistent with previous findings showing lack of *hbl-B* expression [38]. Another interesting observation was that the *hbl*-II locus is flanked by ORFs with homology to recently identified proteins important to replication of the *B. anthracis* virulence plasmid pXO1 [31], which may indicate that this copy is located on a plasmid (Figure 4A). We labeled these as ORFs 14 and 16 in Figure 4A, using the annotations of the corresponding pXO1 genes. PCR using two primer pairs that anneal in the up- or downstream region of the *hbl*-II locus (Table S1) verified the presence of the second Hbl locus in *B. cereus* ATCC 10876 (Figure 4B).

Sequence comparisons of the individual Hbl proteins showed them to be highly homologous to their corresponding paralogs (*i.e.,* L_2_ of *hbl*-I with L_2_ of *hbl*-II, etc.) (Table S2). Although the Hbl-L_2_ paralogs share 77% identity, the L_2_ protein of the *hbl-*II locus has no apparent signal sequence (not shown) and thus it may not be secreted if expressed. *B. cereus* ATCC 10876 also harbors all three genes encoding for NheA, NheB, and NheC (VBlBacCer12024_2764–2766) (not shown). Thus, this strain has the genetic material to produce three tripartite enterotoxins.

### Hbl of *B. cereus* ATCC 10876 is Expressed during Late Exponential Growth Phase

**Figure 5:**
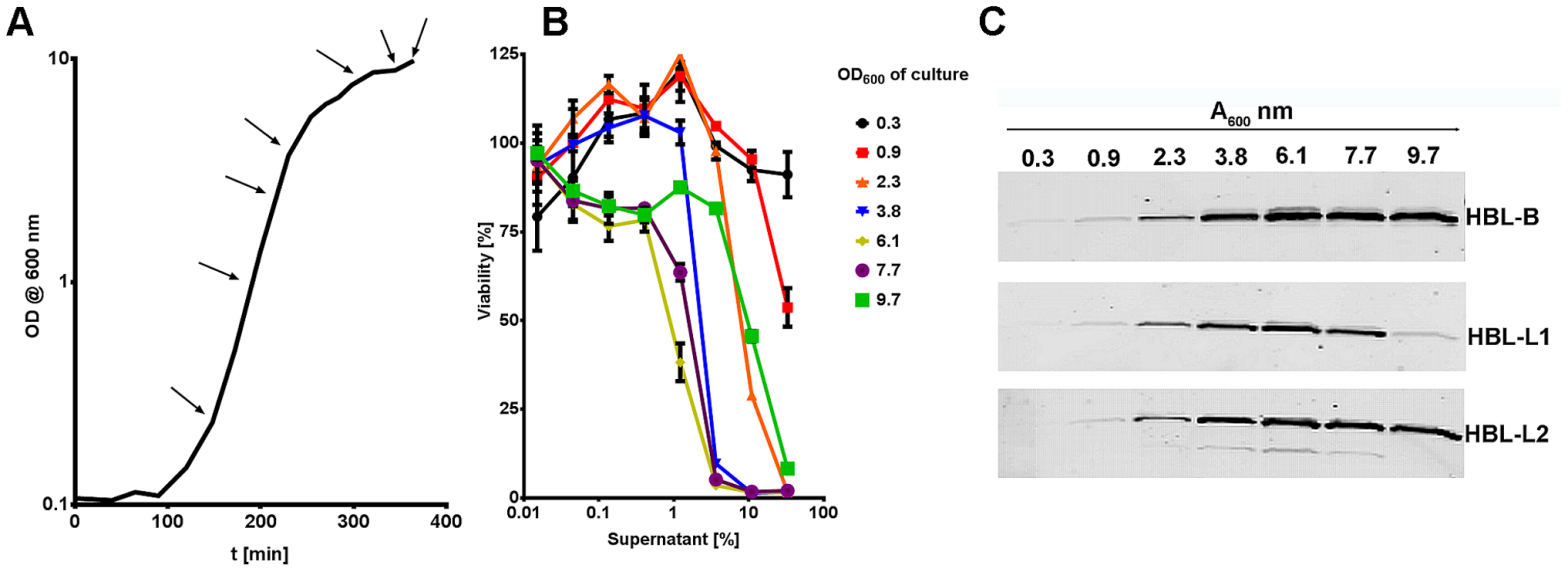
Expression profile of Hbl proteins in *B. cereus* ATCC 10876. (A) Growth measurements (OD at 600 nm) of *B. cereus* ATCC 10876 grown in Brain Heart Infusion broth over time. Arrows indicate points at which samples were withdrawn for analyses performed in (B) and (C). (B) Viability assessment of macrophages incubated for 90 min with *B. cereus* ATCC 10876 sterile, serially-diluted culture supernatants. (C) Western blot analysis of growth-phase dependent expression and accumulation of Hbl toxin components B, L_1_, and L_2_ of *B. cereus* ATCC 10876 in sterile culture supernatants. Toxin proteins were detected using mouse serum containing antibodies raised against single toxin components.

To assess the expression of Hbl over time, we sampled culture supernatants derived from *B. cereus* ATCC 10876 at different times during growth (Figure 5A) and tested for their cytotoxic potential in an *in vitro* assay. Consistent with the growth-dependent activity of the PlcR regulator [39], the highest cytotoxicity was observed for supernatants derived from bacteria grown to mid- to late logarithmic growth phase (A_600_ between 3.8 and 7.7) (Figure 5B). Similarly, all three toxin components were highly expressed during these growth phases (Figure 5C). Interestingly, Hbl component L_1_ appeared to be susceptible to degradation at late stationary phase (Figure 5C). The lower concentration of Hbl-L_1_ in the culture supernatant at the A_600_ of 9.7 was also reflected in the lower toxicity observed for culture supernatants harvested at this absorbance (Figure 5C). These results show that in *B. cereus* ATCC 10876 all three Hbl toxin components are strongly expressed during bacterial growth and that the cytotoxicity profile correlates with Hbl expression. The decrease in L_1_ protein and cytotoxicity at an OD of 9.7 probably reflects the expression at this time of secreted proteases able to degrade toxin proteins.

### Hemolysin BL Contributes to Cytotoxicity *in vitro* and *in vivo*

To investigate the contribution of Hbl to cytotoxicity, we generated a markerless deletion strain using a previously described method that is based on Cre-lox recombination [30], [31]. The resulting strain was deleted for the entire *hbl*-I locus, including the fourth gene, *hbl-B*. Because the L_2_ protein present in the *hbl*-II locus lacks a signal peptide, we assumed that this locus could not contribute to cytotoxicity of bacterial supernatants since all three Hbl toxin components have to be secreted to elicit a toxic effect, although we cannot exclude that it is released upon bacterial lysis.

**Figure 6:**
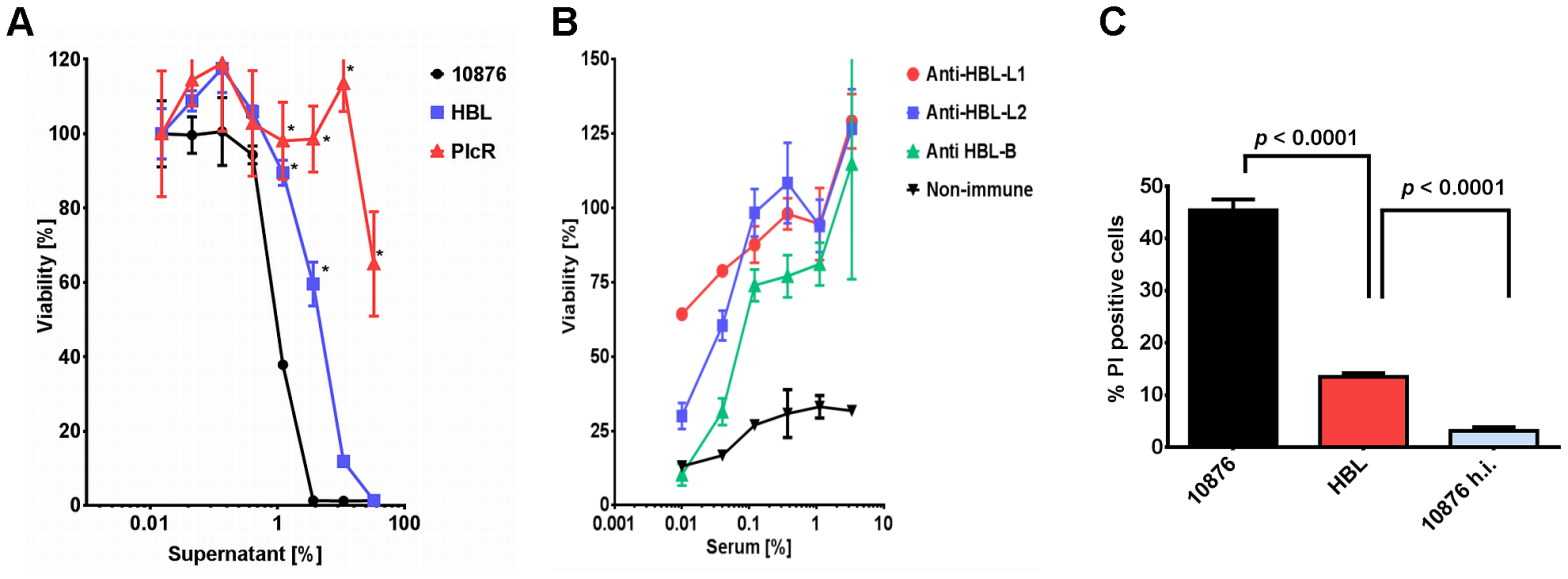
Hbl is the major toxin causing cell death in vitro and in vivo. (A) Viability assessment of macrophages incubated for 90 min with sterile, serially-diluted culture supernatants of *B. cereus* ATCC 10876 and its Hbl and PlcR mutants. Asterisks indicate significant differences in macrophage viability relative to the wildtype (ATCC 10876). (B) Viability assessment of macrophages incubated for 90 min with 2.5% of *B. cereus* ATCC 10876 culture supernatants in the presence of serially-diluted anti-Hbl toxin component or non-immune (control) serum. (C) Flow cytometric assessment of the percentage of propidium iodide-stained cells isolated from the peritoneal cavity of mice (n = 4/group) 45 min after injection with 500 µl of diluted culture supernatants of either *B. cereus* ATCC 10876, its Hbl deleted mutant, or the heat-treated supernatant of *B. cereus* ATCC 10876.

The toxic potential of culture supernatant derived from the Hbl deletion strain grown to early exponential phase (A_600_ of 6.13) was compared to that of wildtype-derived supernatants. We found that toxicity towards macrophages was significantly decreased for the Hbl mutant (Figure 6A), and EC_50_ values were about 4-fold higher when compared to wildtype supernatants. Attenuation did not reach the levels observed for PlcR mutant samples, implying that Hbl is not the only PlcR-dependent toxin causing macrophage lysis.

We additionally tested the ability of mouse anti-Hbl sera to neutralize toxin activity. Macrophages challenged with a lethal dose of *B. cereus* ATCC 10876 culture supernatants were protected when incubated with increasing amounts of serum containing antibodies specific to any of the three Hbl toxin components (Figure 6B). Taken together, these results show that *in vitro*, Hbl is the major cytotoxic protein secreted by *B. cereus* ATCC 10876.

Next, we evaluated the role of Hbl in toxicity *in vivo* using the intraperitoneal toxicity assay. Similar to *in vitro* results, supernatants derived from the Hbl deletion strain were about 3-fold less potent than wildtype-derived supernatants (Figure 6C). However, supernatants derived from the Hbl deletion strain were still significantly more toxic when compared to the heat-treated control supernatants, indicating that Hbl is not the only toxin killing cells *in vivo*.

### Binding of Tripartite Toxin Components to the Eukaryotic Cell Surface

**Figure 7:**
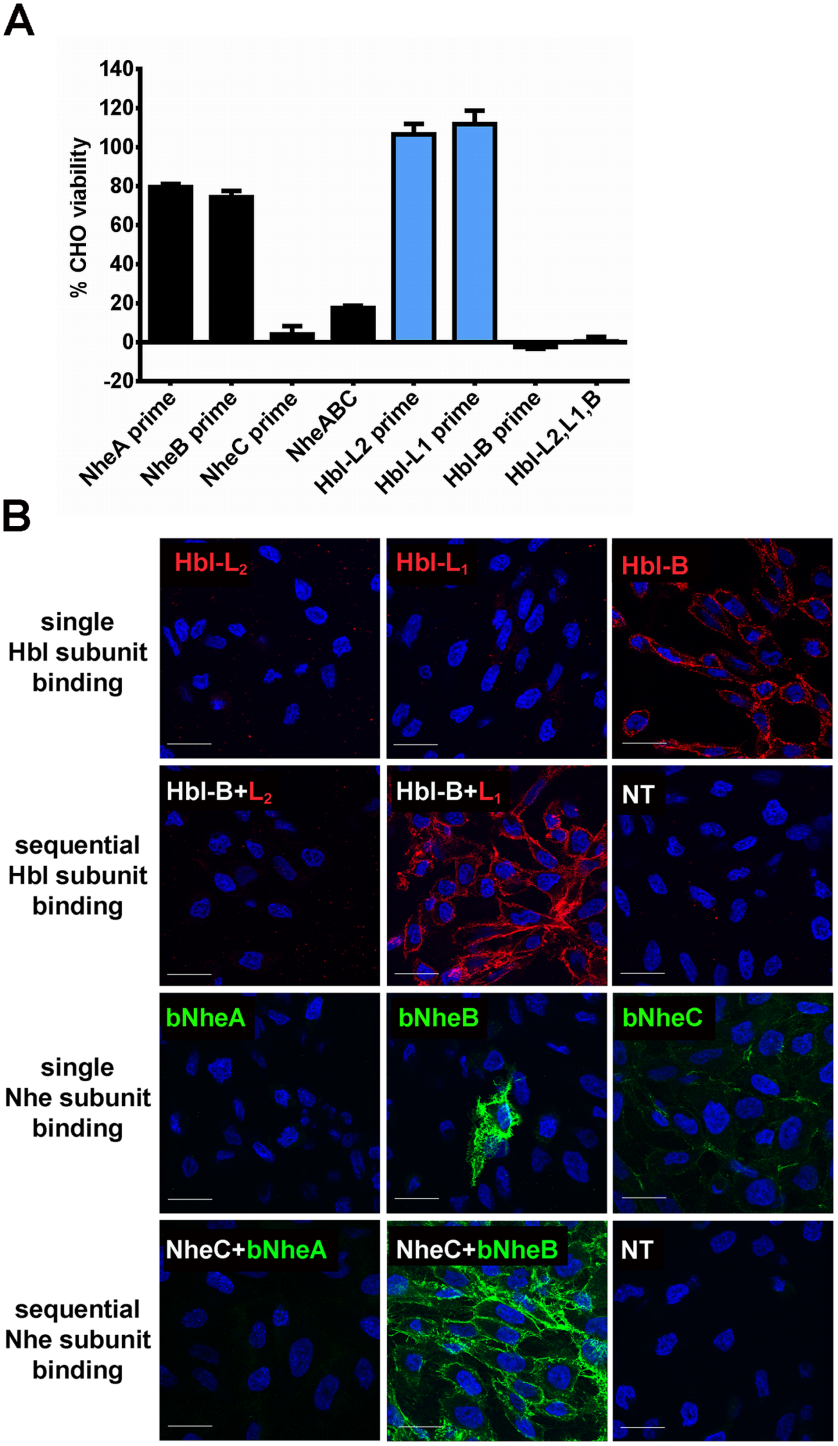
Sequential binding of toxin components to cells. (A) Viability of CHO cells primed for 15 min with either one of the three Nhe (black bars) or Hbl (blue bars) toxin components (5 nM for Hbl toxins, 3% final concentration of recombinant Nhe toxin-containing sterile culture supernatants), followed by washes and challenge with the two complementary components for 30 min (“prime”), or treated with a mixture of all three proteins (A,B,C or L_2_,L_1_,B). Viability is shown relative to control wells treated with the two complementary challenge components only. (B) Confocal microscopic evaluation of toxin component binding to CHO cells. DAPI-stained nuclei are shown in blue. Cell-bound Hbl toxin subunits were detected using antibodies raised against the specific subunit, followed by a secondary, Alexa-Fluor 594 labeled antibody (upper two rows). The specific protein detected is indicated by a red label. Binding of (biotinylated, indicated by a “b” in the toxin name) Nhe toxin subunits was detected by using streptavidin labeled with Alexa Fluor 488 (lower two rows). The detected protein is shown is indicated by a green label. NT samples represent cells not treated with toxin, incubated with the Alexa Fluor labeled antibody alone. Bar represents 20 µm.

To clarify which toxin component represents the cell binding moiety of Hbl, we performed priming studies. Prior studies include conflicting results as to the cell binding protein of the *B. cereus* Nhe tripartite cytotoxin [21], [22]. Because our *B. anthracis* expression system enabled the successful production of all three Nhe toxin proteins, we included these in our studies. CHO cells were first primed with one toxin component, and the other complementary components were added only after extensive washing to remove unbound protein. Because lysis by Hbl and Nhe require all three toxin proteins, cell lysis occurring in this protocol demonstrates that the first toxin protein had bound to the cell membrane. For the Hbl toxin, we observed cell lysis only when we primed with Hbl-B, but not with Hbl-L_1_ or Hbl-L_2_ (Figure 7A). Similarly, priming cells with NheC, which shares 25% protein identity with Hbl-B, resulted in cell lysis after addition of NheA and NheB. We consistently observed some cell killing when cells were incubated for longer periods with either a combination of NheA and NheB, or with Nhe B and C; however, only priming of cells with NheC followed by challenge with NheA and B resulted in killing of nearly 100% of cells (Figure 7A).

For visualization of toxin protein binding to cells we performed immunofluorescent staining of cell-bound toxin proteins using either antibodies (for Hbl toxin components) or fluorescently labeled streptavidin (for biotinylated Nhe toxin proteins) followed by confocal microscopy. Consistent with results obtained for the cell priming study, Hbl-B was the only Hbl component that bound to CHO cells (Figure 7B). Furthermore, subsequent addition of either Hbl-L_1_ or Hbl-L_2_ followed by their specific antibody-based detection showed that only Hbl-L_1_ associates with Hbl-B (Figure 7B). Thus, for Hbl, the order of assembly on the cell surface is B, L_1_, and finally L_2_.

Similar binding studies with recombinant Nhe toxins showed that NheC was the component that consistently associated with cells, whereas NheA and NheB did not (Figure 7B). Interestingly, about 5% of cells incubated with NheB showed very bright fluorescence, indicative of binding of this toxin component to surface of a subpopulation of the cells (Figure 7B). These cells appeared as healthy as neighboring, non-binding cells, and it was not apparent from visual inspection how they differed. We next performed sequential binding experiments by priming cells with unconjugated NheC, followed by incubation with biotinylated NheB or NheA. Binding was then detected with conjugated streptavidin. We detected binding of only NheB, but not NheA to NheC, indicating that similar to the Hbl toxin, Nhe toxin components associated with the cell surface by ordered binding that begins with NheC binding to all cells, followed by NheB, and presumably then NheA, although this last step was not demonstrated in this experiment (Figure 7B).

In summary, our results indicate that the cell binding subunits for the Hbl and Nhe toxin are Hbl-B and NheC, respectively, and that cell lysis induced by both toxins occurs by sequential binding of toxin components initiated by Hbl-B or NheC, followed by the ordered recruitment of Hbl-L_1_ and Hbl-L_2,_ and NheB followed by NheA.

### Nhe and Hbl Toxin Components Cannot Complement Each Other

**Figure 8:**
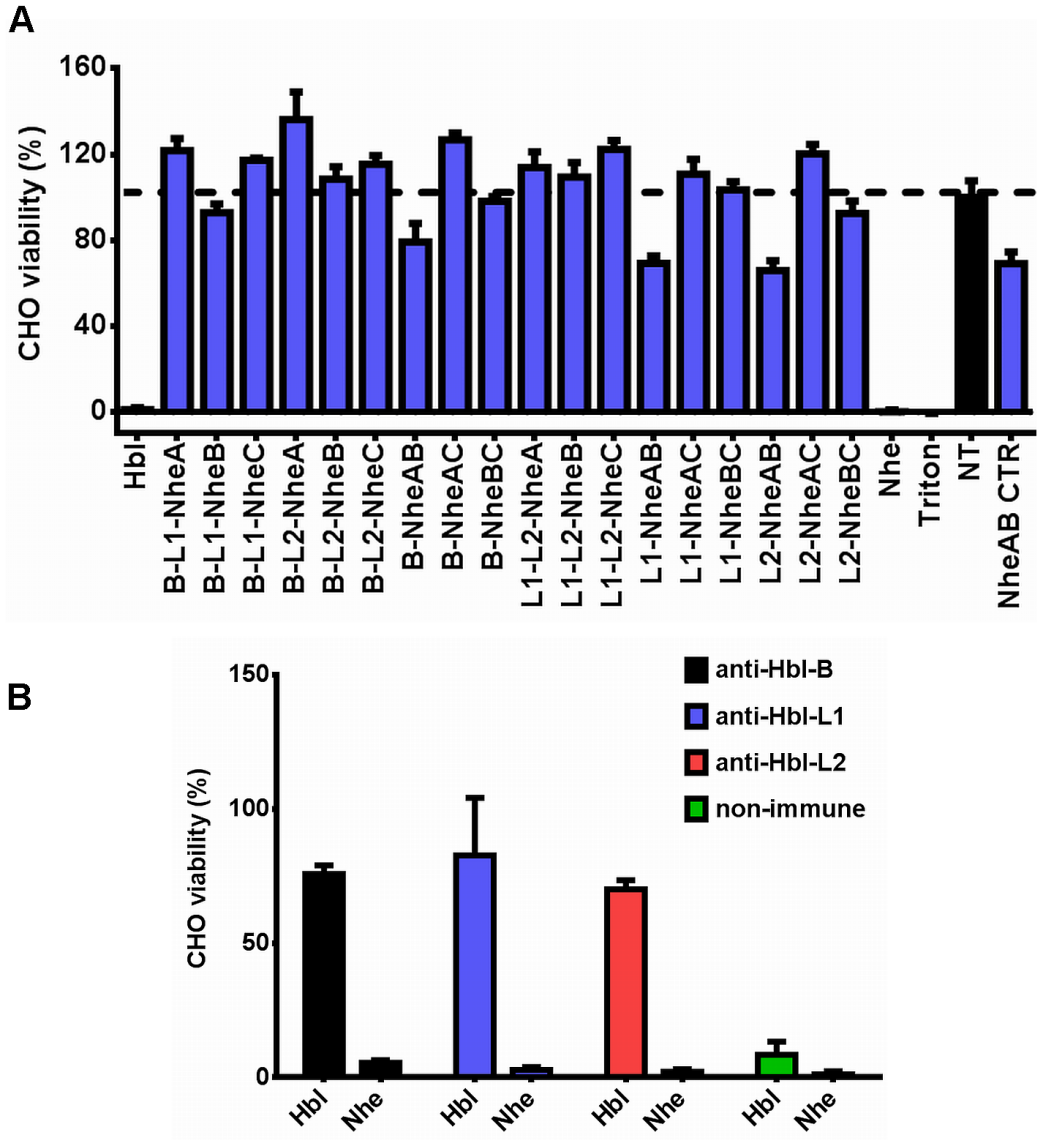
Nhe and Hbl toxin components are not interchangeable. (A) CHO cells were incubated for 30 min with different combinations of Hbl and Nhe toxin components (5 nM for Hbl toxins, 3% final concentration of recombinant Nhe toxin-containing sterile culture supernatants) as indicated. Cells treated with 1% Triton-X-100 or PBS (NT) served as positive or negative control, respectively. An additional control received NheA and NheB components only (last column on the right). The 100% viability mark is indicated by the dotted line. B, Hbl-B; L1, Hbl-L_1_; L2, Hbl-L_2_. (B) Viability assessment of CHO cells incubated with Nhe or Hbl toxins for 30 min in the presence of anti-Hbl toxin component or non-immune (control) serum.

The relatively high sequence similarities of the Nhe and Hbl toxin proteins, which range from 18–40% [16], and the fact that some strains harbor and express both toxins simultaneously [17], [18] prompted us to investigate whether Nhe and Hbl toxin subunits can complement one another. We added Nhe and Hbl toxin proteins to CHO cells in all 20 possible combinations and monitored for cell viability. Because we consistently observed some toxicity when cells were incubated with a mixture of the two components NheA and NheB alone, we included these as an additional control. Our results show that while incubation of cells with either all three Nhe or all three Hbl components resulted in nearly 100% cell death, any of the other 18 combinations were non-toxic (Figure 8A). The slightly reduced viability observed for cells challenged with either Hbl toxin component in combination with NheA and NheB was caused by the latter two toxin components, evidenced by comparable loss of viability for cells incubated with NheA plus NheB alone (Figure 8A). Furthermore, although some Nhe and Hbl proteins share sequence homology, antibodies raised against single Hbl toxin components failed to protect from Nhe-induced cell death (Figure 8B) and only weakly recognized Nhe subunits (not shown). Thus, our results indicate that although *B. cereus* strains can harbor and express two toxins that are similar in structure and sequence, the single components of both toxins are not able to complement or substitute for one another to form an active toxin.

## Discussion

We initiated this study to identify the toxin(s) responsible for a very rapid cell death we observed during an earlier study that investigated the role of the regulator PlcR in protein expression and toxicity of *B. anthracis* secreted proteins [36]. Here, we found that the *B. cereus* induced cell toxicity *in vitro* was independent of cell type and origin, but dependent, to some extent, on the presence of the regulator PlcR. Furthermore, in our intraperitoneal toxicity model, we found that *B. cereus* secreted proteins can kill approximately 50% of cells within 10 min post-injection. Efforts to address cytotoxicity using *B. cereus* bacteria in this model failed because of the infiltration of large numbers of neutrophils within hours post-injection, which masked the number of killed cells (not shown). Using size fractionation and anion exchange coupled with genetic and antibody protection studies, we identified the tripartite toxin hemolysin BL as the responsible toxic entity. Hbl is not only one of four toxins associated with *B. cereus* food poisoning symptoms, but in addition to other PlcR regulated proteins it also contributes to the symptoms associated with endophthalmitis [40], [41], a severe infection of the eye that often results in blindness [42].

We confirmed the growth-phase dependent expression of Hbl in *B. cereus* ATCC 10876, as described for PlcR-controlled genes [43]. Furthermore, analysis of the genome revealed that *B. cereus* ATCC 10876 harbors two copies of *hbl* genes. Because the *hbl-II* locus is surrounded by genes similar to genes previously associated with replication of the *B. anthracis* plasmid pXO1 [31], we suspect that this region of the genome is actually plasmid sequence that was not properly annotated as such, as this particular strain, which is also known as *B. cereus* 569, contains several plasmids [44].

A remarkable feature of Hbl is the requirement of three toxin subunits (B, L_1_, L_2_) for pore formation. Many bi-component bacterial toxins have been described, such as the classic AB toxins that are composed of a cellular binding and an enzymatic effector subunit. The latter subunit can either be encoded by a separate gene, or both components can be synthesized as one polypeptide [45], [46]. The Cytolethal Descending Toxins of some gram-negative pathogens are tripartite toxins that also function in an AB-type manner. Some bacteria also express bi-component pore-forming toxins, such as staphylococcal leukocidins that cause lysis of cells of the leukocytic lineage [47]. However, to our knowledge, Hbl and the structurally similar Nhe toxin of *B. cereus* are the only known pore-forming toxins described to date that require the assembly of three subunits on the cellular membrane to induce lysis.

To date, little is understood about the mechanisms by which the Hbl or Nhe toxin subunits assemble to create a pore. Structurally, the binding subunit of Hbl, Hbl-B, is very similar to the hemolysin E enterotoxin of *E. coli* [24], a single-component hemolysin which forms a dodecameric pore on the eukaryotic membrane [27]. Thus, it is likely that Hbl-B as well as NheB and NheC, which were predicted to have a similar structure, also form oligomeric structures that resemble hemolysin E. Indeed, NheB has recently shown to oligomerize in the presence of dodecyl maltoside [48], a detergent that has been successfully used to solve the hemolysin E dodecameric structure [27].

To gain further insight into the toxin mechanism, we used both priming studies and microscopy to address which of the three Hbl toxin subunits binds to the surface of CHO cells. Previous studies presented evidence that Hbl-B is the binding moiety of Hbl [19]. However it was later suggested that both L_1_ and L_2_ can independently bind to erythrocytes [20]. Our results clearly showed that Hbl-B is the only protein able to associate productively with the cell membrane, and this binding is followed by the ordered recruitment of first L_1_ and then L_2_.

Sequential binding of multi-component toxins has been described before for staphylococcal toxins [49] and was also suggested for the Nhe toxin [22], [50], although for the latter toxin the model proposed suggested that both NheB and NheC are capable of associating with the cell surface [21], [22]. Using our *B. anthracis* system, we were able to receive high yield expression of Nhe toxins, allowing us to tested the binding of single Nhe toxin components to the surface of CHO cells. Similar to a previous report [22] we did not detect NheB binding using priming assays, and only priming with NheC followed by challenge with the complementary components resulted in lysis. Similarly, microscopic evaluation of cells incubated with NheC showed homogenous association of this subunit with all cells. In contrast, NheB only bound to very few cells, but to a much higher extent and very consistently using different protein preparations. We are uncertain about the physiological consequences of this binding. A more recent study suggested that at higher concentrations, NheB attaches unspecifically to Vero cells, which may result in an inability to further form active complexes upon binding of NheC and NheA [23]. Our findings parallel these results. We show that although NheB binds to some cells (as shown by microscropy), no active toxin complex is assembled, evidenced by the survival of NheB primed cells subsequently incubated with NheA and NheC (as shown in the priming assays). In summary, our data using CHO cells indicate the sequential binding of NheC, followed by NheB, and lastly NheA.

Some *B. cereus* strains can simultaneously express both Nhe and Hbl [17], [18] toxins, which share up to 40% sequence identity and probably have similar structures [16]. Thus, we wondered whether the subunits of these two toxins are interchangeable, so they could complement one another and assemble into heterologous, functional pores. However, testing all 20 possible combinations of the six subunits showed that only the homologous tripartite combinations elicited cytotoxicity. The toxicity we observed when incubating cells with toxin combinations that contained both NheA and NheB was caused by these two toxins alone. A study that investigated the pore forming ability of Nhe toxin components found that NheA plus NheB alone induced large conductance channels in pituitary (GH_4_) cells that were comparable to channels observed when cells were incubated with all three toxin subunits. Interestingly, smaller channels were observed for epithelial (Vero) cells, indicating the presence of a cell-specificity. We also found that Hbl-specific antibodies are not able to neutralize Nhe toxin action, paralleling the findings of our complementation study. Thus, although similar in sequence and structure, each tripartite toxin has a unique assembly mechanism and the subunits can only interact with subunits of the same toxin.

In conclusion, our work provides further evidence that the diarrhea-associated *B. cereus* tripartite enterotoxins Hbl and Nhe are highly potent cytotoxins that assembly on the eukaryotic membrane in an ordered, sequential manner, similar to that described for other bi-component toxins or the membrane attack complex of the innate immune system [51]. Although Nhe and Hbl appear to have a similar assembly mechanism, these two toxins, which can be simultaneously expressed and even may have arisen by gene duplication, are incompatible. Furthermore, in light of previous publications, our Nhe toxin data imply the existence of differences in toxin assembly orders that are dependent on the cell type, further differentiating the Hbl toxin, which appears to act on all cell types in a similar manner, from the closely related Nhe toxin.

## Supporting Information

Table S1 (PDF)

Table S2 (PDF)
