# Supplementary material for: The Bacillus cereus Hbl and Nhe Tripartite Enterotoxin Components Assemble Sequentially on the Surface of Target Cells and Are Not Interchangeable
Source: PLoS One. 2013 Oct 18;8(10):e76955. doi: 10.1371/journal.pone.0076955 (PMC3799921; doi:10.1371/journal.pone.0076955)
Supplement: Table S1 — (PDF) [file pone.0076955.s001.pdf]

Table S1: Primers used in this study

Name	5'- 3' sequence	Purpose
pHbl_ up1<br>pHbl_up1	tgtttggttcaattttatggatca<br>ttggcattgcgagtgtattg	PCR of *hbl*-2 upstream region
pHbl_down1<br>pHbl_down2	ttggaaaacattaagagcagatg<br>tcgcaatcatagatcgtttcc	PCR of *hbl*-2 downstream region
Hbl-LF1<br>Hbl-LF2	*ccccgaattc*ggaagctttgcagcttttt<br>*ccccactagt*ccgctctctttgtaaactgga	Hbl genetic deletion, upstream region
Hbl-RF1<br>Hbl-RF2	*ccccgaattc*cgaagtaattggagctggaga<br>*ccccactagt*acaggaggttcccagtaccc	Hbl genetic deletion, downstream region
rHblBfw<br>rHblBrv	*aacat*atgttggaggaaaatgaaatgat<br>*AAGGATCC*ttattagtgatggtgatgatgatgtttttgtggagtaacagtttcca	Cloning of HblB
rHblL1fw<br>rHblL1rv	*ggatta*atgaaaaaatttccattcaaagtactaac<br>*aaggatcc*ttattagtgatggtgatgatgatgctcctgtttaaaagcaatatcttttg	Cloning of Hbl-L_1
rHblL2fw<br>rHblL2rv	*aacat*atgaaaactaaaataatgacaggattt<br>*aaggatcc*ttattagtgatggtgatgatgatgaaatttatatacttgttcttcaaggtaactta	Cloning of Hbl-L_2
rNheAfw<br>rNheArv	*aacatatg*aaaaagactttaattacagggttattg<br>*aaggatcc*ttattagtgatggtgatgatgatgatgtacttcaacgtttgtaacgtaatc	Cloning of NheA
rNheBfw<br>rNheBrv	*aacat*atgacaaaaaaaccatataaagtaatgg<br>*aaggatcc*ttattagtgatggtgatgatgatgtgctttttcgtgtctactactttaatatc	Cloning of NheB
rNheCfw<br>rNheCrv	*aacat*atgcagaaacgattttataaaaaatg<br>*aaggatcc*ttattagtgatggtgatgatgatgctttgccacaccttcatgtaatt	Cloning of NheC

Italicized letters indicate restriction recognition sites for *Bam*HI, *Ase*I, *Nde*I, *Eco*RI, or *Spe*I. Underlined letters indicate histidine tags and stop codons.
