# Supplementary material for: The Bacillus cereus Hbl and Nhe Tripartite Enterotoxin Components Assemble Sequentially on the Surface of Target Cells and Are Not Interchangeable
Source: PLoS One. 2013 Oct 18;8(10):e76955. doi: 10.1371/journal.pone.0076955 (PMC3799921; doi:10.1371/journal.pone.0076955)
Supplement: Table S2 — (PDF) [file pone.0076955.s002.pdf]

Table S2: Summary of Hbl protein sequence identities

		***Hbl*-I**			
***Hbl*-II**		L2 (4070)	L1 (4071)	B (4072)	B (4073)
	L2 (1069)	77 %			
	L1 (1070)		86 %		
	B (1071)			70 %	68 %
